# Effect of intracanal medicaments used in endodontic regeneration on the push-out bond strength of a calcium-phosphate-silicate-based cement to dentin

**DOI:** 10.12669/pjms.342.14630

**Published:** 2018

**Authors:** Elif Aybala Oktay, Seyda Ersahan, Selcuk Gokyay

**Affiliations:** 1 Elif Aybala Oktay, Department of Restorative Dentistry, Faculty of Dentistry, Health Sciences University, Ankara, Turkey; 2 Seyda Ersahan, Department of Endodontics, Faculty of Dentistry, Medipol University, Istanbul, Turkey; 3 Selcuk Gokyay, Department of Endodontics, Faculty of Dentistry, Istanbul University, Istanbul, Turkey

**Keywords:** Bond strength, Endosequence Root Repair Material (ERRM), Intracanal medicaments, Push-out test, Regenerative endodontic treatment

## Abstract

**Objective:**

To evaluate the effects of various endodontic regeneration agents on the push-out bond strength of Endosequence Root Repair Material (ERRM) to root-canal dentin.

**Methods:**

Fifty single-rooted human teeth were selected and instrumented to obtain a standard internal diameter of 1.5 mm. Specimens were randomly divided into four experimental groups and treated with an intracanal medicament [calcium hydroxide (CH), double antibiotic paste (DAP), triple antibiotic paste (TAP), TAP with amoxicillin (mTAP)] and a non-treated control group. Medicaments were removed after three weeks, and ERRM was applied to all specimens. The coronal portion of each root was then sliced into 2-mm-thick parallel transverse sections (2 slices per tooth, n=20 slices per group), and a push-out test was used to measure the bond strength of ERRM to dentin. Data were analyzed using Bonferroni-corrected Mann-Whitney tests, with the level of significance set at p<0.05.

**Results:**

The push-out bond strength of the CH group was significantly higher than that of the TAP, DAP and mTAP groups (*p*< 0.005). Furthermore, the bond strength of the control group was higher than the bond strength of both the DAP and mTAP groups.

**Conclusion:**

The use of CH in clinical practice may help improve the adhesion of ERRM to dentin.

## INTRODUCTION

Regenerative endodontic protocols generally involve disinfection of the root canal followed by the introduction of a blood clot and/or stem/progenitor cells into the root-canal space, which is then restored with a microorganism-impregnable material, allowing tissue repair and further root maturation.^1^

Triple antibiotic paste (TAP), which is a mixture of metronidazole, ciprofloxacin and minocycline, is the medicament most widely used in endodontic regeneration.^2^ Double antibiotic paste (DAP), which includes only metronidazole and ciprofloxacin, has also been successfully used as a canal dressing and has been suggested as a substitute for TAP to avoid the discoloration caused by minocycline.^2^ Recently, a modified triple antibiotic paste (mTAP) comprised of metronidazole, ciprofloxacin and amoxicillin has been suggested as another alternative.^3^ Calcium hydroxide [Ca(OH)_2_, CH] has also been used to disinfect the canal during endodontic regeneration.^4^ Although both antibiotic pastes and CH have regenerative effects, they can alter the chemical structure of dentin and the mineral content of dentin surfaces.^5^ If the dressing is not thoroughly removed from the root canal, it can interfere with the bond between root dentin and the root-canal sealant applied prior to the final restoration.^6^

The strength of the bond between the root canal sealant and dentin plays an important role in the clinical success of endodontic treatment in terms of maintaining an adequate seal and minimizing the risk of restoration detachment during treatment and masticatory functioning.^7^ The inability of the barrier material to resist displacement forces, including the condensation of subsequent restorative materials and the mechanical loads of occlusion,^8^ may also have a negative affect on scaffold formation. Push-out bond-strength test has been usually used in measuring the adhesive strength of root-canal sealers, because the test is easy to reproduce and interpret, and it provides a realistic assessment of bond strength to dentin, even at low levels.^9^ The material most commonly used to create a barrier is mineral trioxide aggregate (MTA), which was the first calcium silicate-based cement patented for endodontic applications. MTA has shown good adhesion, especially when used in regenerative endodontic procedures.^10^ But it has certain drawbacks, including long setting times, difficulty with manipulation, limited resistance to washout before setting, and the possibility of staining tooth structure.^10^ Endosequence BC Root Repair Material (ERRM; Brasseler USA, Savannah, GA, USA) is a combination of calcium silicates, zirconium oxide, tantalum oxide, calcium phosphate monobasic and filler agents. Studies have shown ERRM to be antibacterial and biocompatible.^11^ Previous studies have also reported ERRM to be equivalent to MTA in terms of sealing ability.^12^ However, there is no information available regarding the effects of intracanal medicaments on the ability of calcium-phosphate-silicate-based cement to adhere to root-canal dentin. Therefore, this study aimed to evaluate the effects of four intracanal medicaments widely used in regenerative endodontics on the push-out bond strength of the ERRM to root canal dentin. The null hypothesis tested was that the intracanal medicament used would not affect the push-out bond strength of ERRM.

## METHODS

### Sample selection and preparation

This study was conducted using 50 single-rooted human teeth stored in 0.5% chloramine T at 4°C for a maximum of two weeks before use (Institutional Ethics Committee number: B30.2.001.00.89). Teeth were decoronated to a standardized root length of 15 mm. Working lengths were established, and root canals were prepared to a size #50, 0.05 taper (F5) using ProTaper rotary instruments (Dentsply Maillefer, Ballaigues, Switzerland). Peeso reamers (Mani Inc, Tochigi, Japan) ranging in size between #1 and #6 were introduced into the canals to obtain a standard internal diameter of 1.5 mm. Root canals were irrigated with 2 mL 1.5% sodium hypochlorite (NaOCl) between instruments and with 20 mL 1.5% NaOCl for five minutes following instrumentation. Canals were then rinsed with 20 mL saline and dried with paper points. Teeth were then randomly divided into four experimental groups and a control group (n=10 teeth per group). Experimental groups were treated with either CH, DAP, TAP, or mTAP as an intracanal medicament, whereas no dressing was used for the control group.^13^

### Preparation and placement of intracanal medicaments

Intracanal dressings were prepared as follows:^14^

### CH group

CH powder (Kalsin, Aktu Tic, Izmir, Turkey) was mixed with distilled water (2:1powder/liquid ratio).

### DAP group

Equal amounts (250 mg tablets) of metronidazole (Eczacibasi, Istanbul, Turkey) and ciprofloxacin (Biofarma, Istanbul, Turkey) were mixed with distilled water (0.50 mL) (1 g/mL).

### TAP group

Equal amounts (167 mg tablets) of metronidazole, ciprofloxacin and minocycline (Ratiopharm, Ulm, Germany) were mixed with distilled water (0.50 mL) (1 g/mL).

### mTAP group

Equal amounts (167 mg tablets) of metronidazole, ciprofloxacin and amoxicillin (Bilim, Istanbul, Turkey) were mixed with distilled water (0.50 mL) (1 g/mL).

A #40 lentulo spiral was used to place the paste in the root canal. No dressing material was applied to the canals in the control group. Access cavities were covered with a temporary filling material and the specimens were stored at 37°C/100% humidity for 3 weeks to simulate in vivo conditions.^3^ After three weeks, the canals were gently irrigated with 20 mL 17% EDTA followed by 10 mL saline and dried with paper points. ERRM (Brasseler USA, Savannah, GA, USA) was injected into each canal and compacted with a plugger to a length of 6 mm below the cemento-enamel junction. Specimens were then wrapped in gauze soaked in distilled water and stored at 37°C/100% humidity for one week to allow the ERRM to set completely.

### Push-out Bond Strength Testing

**Fig.1:**
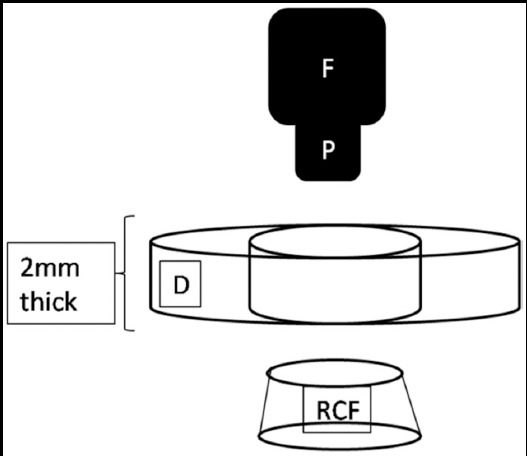
Scheme of the specimen positioned on the apparatus for load application in the universal testing machine (F: force; P: plugger; RCF: root canal filling; D: diameter)

The coronal portion of each root was sectioned perpendicularly to its long axis into 2 2.00 ± 0.05 mm-thick slices using a water-cooled diamond saw to obtain 20 slices per group. The push-out bond strength was performed using a universal-testing machine (Shimadzu AG-1; Shimadzu Corp, Tokyo, Japan). A 1.20-mm diameter cylindrical stainless-steel plunger applying a constant compressive load at a crosshead speed of 0.5 mm/min^-1^ was positioned in such a way that it only contacted the filling material and avoided contact with the dentin. Also, the disk samples were positioned to allow the plunger to move in an apical to coronal direction, which resulted in the displacement of the filling material toward the larger diameter coronal aspect of the disk (Fig.1). This method ensured the alignment of the specimen in an accurate and reproducible manner and also maintained the plunger centralized and avoided its contact with the dentin wall when the material was pushed and dislodged from the dentin wall. The maximum load applied before failure was recorded in Newtons and converted to megapascals (MPa).^14^

### Failure analysis

Following push-out testing, all specimens were examined under a stereomicroscope (SZ61; Olympus, Tokyo, Japan) at 40× magnification to evaluate the failure type. Three types of failure were categorized: adhesive failure (failure was at the sealer/dentin interface), cohesive fracture (failure was entirely within the sealer), and mixed (failure in both the sealer and dentin).^14^

### Statistical analysis

Bond-strength data were evaluated using a Kruskal-Wallis test, and pair-wise *post-hoc* comparisons were performed using a Bonferroni-corrected Mann-Whitney *U* test. Categorical data were compared using chi-square and Fisher’s exact tests. All statistical analysis was performed using the MedCalc Statistical Software package version 12.7.7, with a *P-*value of <0.05 considered statistically significant.

## RESULTS

**Fig.2:**
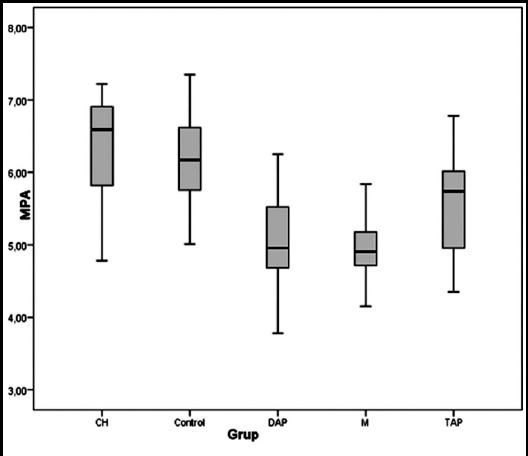
Box plots illustrating mean, minimum and maximum values and variance of push-out bond strength data for each experimental group

Mean push-out bond strength values of the control, CH, TAP, DAP and mTAP groups were 6.20±0.69, 6.35±0.68, 5.62±0.66, 5.03±0.63 and 4.99±0.46 MPa, respectively (Kruskal Wallis test, *p*< 0.001) (Fig.2). Multiple paired comparisons showed significant differences between the CH and TAP (*p* = 0.004), CH and DAP (*p*< 0.001), CH and mTAP (*p*< 0.001), TAP and mTAP (*p*=0.001), DAP and control (*p*< 0.001), and mTAP and control (*p*< 0.001) groups (Mann-Whitney with Bonferroni correction, *p*< 0.005). No significant differences were found between the CH and control (*p* = 0.495), TAP and control (*p* = 0.012), TAP and DAP (*p*=0.007), or DAP and mTAP (*p*=0.841) groups. Multiple paired comparisons showed the CH group to have significantly higher bond strength than the TAP, DAP and mTAP groups (Mann-Whitney with Bonferroni correction, *p*< 0.005). Failure analysis showed the predominant failure modes to be cohesive failure for the Control, CH and TAP groups and adhesive failure for the DAP and mTAP groups (Table-I).

**Table-I:** Mean failure modes for each group (Fisher’s Exact test, p< 0.05).

	Adhesive n (%)	Cohesive n (%)	Mixed n (%)	p^1^
TAP	2 (10.0)	12 (60.0)	6 (30.0)	<0.001
DAP	10 (50.0)	7 (35.0)	3 (15.0)	
mTAP	10 (50.0)	8 (40.0)	2 (10.0)	
CH	0 (0.0)	15 (75.0)	5 (25.0)	
Control	0 (0.0)	14 (70.0)	6 (30.0)	

1 Fisher’s Exact p.

## DISCUSSION

The findings of this study showed intracanal medicaments to affect the bond between ERRM and root dentin. The application of antibiotic pastes resulted in decreases in ERRM bond strength in comparison to untreated controls. Moreover, the application of CH as an intracanal medicament resulted in a non-significant increase in ERRM bond strength in comparison to controls. Thus, the null hypothesis was partially rejected.

The increase in bond strength of ERRM to dentin observed following the application of CH may be the result of either a chemical interaction between CH residue and the sealant material,^15^ or an increase in the frictional resistance and/or micromechanical retention of the sealant.^16^ No previous studies have reported on the effects of intracanal medicaments on the bond strength of ERRM to dentin; therefore, the results of this study were compared with previous studies examining the effects of intracanal medicaments on the bond strength of MTA. There are conflicting reports regarding the effect of residual CH on the bonding capacity of endodontic sealers. Bidar M et al.^17^ found CH improved the marginal adaptation of MTA used as an apical plug, and the authors suggested this could be due to a reaction between the MTA and the residual CH. In contrast to these findings, Guiotti FA et al.^18^ and Topcuoglu HS et al.^16^ determined that prior application of a CH dressing did not significantly affect the push-out bond strength values of MTA. The discrepancies among study findings may be due to differences in the compositions of the sealers tested.

In the current study, the push-out bond strengths of the specimens previously treated with antibiotic medicaments (TAP, mTAP, and DAP) were significantly lower than those of the specimens treated with CH. These results suggest that irrigation is unable to adequately remove residual medicaments containing antibiotics. A previous study comparing the removal of CH and TAP from root canals found minimal amounts of CH residue, which was distributed rather superficially, and contained a smaller amount of material in comparison to TAP, which appeared to have greater retention and deeper penetration, making it more difficult to remove.^6^ In the present study, among the three antibiotic medicaments tested, the TAP group had higher bond strengths than both the mTAP and DAP groups.

The most likely explanation for this is that the bond strength of the sealant to dentin is increased through the chelation of calcium ions in dentin to residual minocycline from TAP.^19^ The results of the present study are inconsistent with those of previous studies conducted using MTA. For example, although Topcuoglu HS et al.^16^ found DAP to have a negative effect on the sealing ability of MTA, which is in line with the present study’s finding that DAP negatively affecting the bond strength of ERRM, the authors of this earlier study also reported CH and TAP to result in similarly high push-out bond strengths, which conflicts with the present study’s finding that CH application resulted in significantly higher bond strengths when compared to TAP application.^16^ Moreover, Dumani A et al.^20^ found the push-out bond strength of MTA to dentin was significantly decreased by TAP application, even though the TAP was removed from canals by irrigation. By contrast, while the present study found TAP application lowered the push-out bond strength of ERRM to dentin, the difference in bond strength was not statistically significant between the TAP and control groups. Given the differences in methodologies and materials between Topcuoglu HS et al.^16^ and Dumani A et al.^20^ and the present study, the differences in findings are unremarkable.

### Methodological considerations

Although endodontic regeneration usually requires minimal or no instrumentation, in this study, root-canal instrumentation was performed in order to standardize the internal dimensions of the root specimens (to 1.5 mm diameter). Following instrumentation, irrigation was performed as recommended by the American Association of Endodontists (AAE) in order to replicate clinical practice.^21^ Accordingly, conventional syringe irrigation was performed using 20 mL 17% EDTA followed by 10 mL saline in order to remove the intracanal medicaments. Although EDTA irrigation has been recommended as a means of clearing away medicament residue, opening dentinal tubules, and exposing various growth factors during endodontic regeneration,^22^ it has also been suggested that EDTA may inhibit the adhesion of hydrophilic materials to dentin by decreasing the wetability of dentin surfaces.^23^ Thus, even though saline was used as a final irrigation in this study, it may not have sufficiently flushed away residual EDTA and increased dentin wetability to allow ERRM, which is hydrophilic by nature, to achieve adequate adhesion to root dentin.^23^ In this regard, one of the most important limitations of this study is that it did not evaluate the canal lumen or the amount of residual medicaments.

Another methodological issue to be noted in connection with the current study has to do with the concentrations of the antibiotic pastes used. Concerns have been raised that the relatively high concentrations of antibiotic pastes used during endodontic regeneration may have negative effects on the mechanical properties of radicular dentin^5^ and either direct or indirect cytotoxic effects on human apical papilla stem cells^24^ and dental pulp cells.^25^ In line with clinical practice, in order to be able to produce a paste with the consistency required for it to be successfully introduced into an infected immature root canal, this study used TAP, DAP and mTAP concentrations of 1 g/mL,^26^ despite the fact that AAE has recommended a much lower concentration of 0.1 mg/mL.^21^ The AAE recommendation is most likely based on the results of laboratory studies that have reported drug concentrations of more than 100 mg/mL to be cytotoxic to SCAPs and dental-pulp stem cells.^26^ While the use of lower concentrations of antibiotic medicaments might also help to minimize their negative effects on the mechanical properties of radicular dentin, the application of such low concentrations in clinical situations is difficult. Additional studies are needed to address this issue.

In order to simulate clinical practice and to minimize the detrimental effects of intracanal medicaments on bond strength, the present study protocol called for medicaments to remain in root canals for three weeks. Although some previous studies have recommended an interval of 2-6 weeks between first and second appointments for endodontic regeneration treatment,^27^ other studies have suggested that exposure to antibiotic pastes for periods of between 4-12 weeks may have a negative effect on the physical structure of dentin,^5^,^27^ leading to demineralization of the dentin matrix and an increased susceptibility to fracture.^5^

Finally, it should be noted that in spite of the manufacturer’s claim that ERRM has a setting time of four hour under normal conditions, an in vitro study by Damas BA et al.^11^ that focused on setting time of root-canal cements found that ERRM specimens stored in 100% humidity had not completely set in 72 hour. In the present study, in addition to storage in 100% humidity, cotton pellets moistened with distilled water were placed over the ERRM material, and specimens were wrapped in gauze soaked in distilled water. All of the ERRM samples were observed to have fully set within one week. In the present study, the effect of intracanal medicaments on bond strength of set ERRM was only evaluated. However in some previous studies, in order to simulate the clinical conditions, the surfaces of the biomaterial were in contact either with blood or phosphate-buffered solution (PBS).^28^,^29^ Enhanced biomineralization of MTA was observed as the formation of an interfacial layer with tag-like structures in those studies.^28^ Furthermore, additional research would be necessary to determine the push-out bond strength for ERRM in the situations similar to clinical conditions.

## CONCLUSION

The push-out bond strength of ERRM to root dentin is significantly higher when CH is used as an intracanal medicament as compared to an intracanal medicament containing antibiotics. These findings suggest that the use of CH in clinical practice may help improve the adhesion of calcium phosphate silicate cement to dentin.
